# Urine Tenofovir Levels Measured Using a Novel Immunoassay Predict Human Immunodeficiency Virus Protection

**DOI:** 10.1093/cid/ciaa785

**Published:** 2020-06-22

**Authors:** Randy M Stalter, Jared M Baeten, Deborah Donnell, Matthew A Spinelli, David V Glidden, Warren C Rodrigues, Guohong Wang, Michael Vincent, Nelly Mugo, Andrew Mujugira, Mark Marzinke, Craig Hendrix, Monica Gandhi, for the Partners PrEP Study Team, Connie Celum, Jared M Baeten, Deborah Donnell, Robert W Coombs, Jairam R Lingappa, M Juliana McElrath, Kenneth H Fife, Edwin Were, Elioda Tumwesigye, Patrick Ndase, Elly Katabira, Elly Katabira, Allan Ronald, Elizabeth Bukusi, Craig R Cohen, Jonathan Wangisi, James D Campbell, Jordan W Tappero, James Kiarie, Carey Farquhar, Grace John-Stewart, Nelly R Mugo, Kenneth Ngure, James D Campbell, Jordan W Tappero, Jonathan Wangisi

**Affiliations:** 1 Department of Global Health, University of Washington, Seattle, Washington, USA; 2 Department of Epidemiology, University of Washington, Seattle, Washington, USA; 3 Department of Medicine, University of Washington, Seattle, Washington, USA; 4 Vaccine and Infectious Disease Division, Fred Hutchinson Cancer Research Center, Seattle, Washington, USA; 5 Division of HIV, ID, and Global Medicine, University of California–San Francisco, San Francisco, California, USA; 6 Toxicology Division, Abbott Rapid Diagnostics, Pomona, California, USA; 7 Center for Clinical Research, Kenya Medical Research Institute, Nairobi, Kenya; 8 Infectious Diseases Institute, Makerere University, Kampala, Uganda; 9 Division of Clinical Pharmacology, Department of Medicine, Johns Hopkins University, Baltimore, Maryland, USA

**Keywords:** PrEP, HIV prevention, urine, immunoassay, ELISA

## Abstract

New tools are needed to support pre-exposure prophylaxis (PrEP) adherence for human immunodeficiency virus (HIV) prevention, including those that enable real-time feedback. In a large, completed PrEP trial, adequate urine tenofovir levels measured using a novel immunoassay predicted HIV protection and showed good sensitivity and specificity for detectable plasma tenofovir.

Use of oral pre-exposure prophylaxis (PrEP) is a highly efficacious strategy for preventing human immunodeficiency virus (HIV) when taken daily [1]. However, adherence to PrEP has proven challenging among many populations [2]. Due to the limited accuracy of self-reported adherence, objective biological adherence metrics, such as measurement of PrEP drug concentrations in plasma, dried blood spots (DBS), and hair, have become valuable tools for appraising recent or cumulative PrEP adherence [3]. However, these methods can be costly and often require shipment of samples to an external laboratory, skilled laboratory personnel, and specialized equipment, making them impractical options for routine use, particularly in resource-limited settings.

Innovative approaches are needed to support PrEP adherence, including those that enable counseling at the point of care (POC). Recently, we reported on the development of a novel antibody with high selectivity for tenofovir (TFV) [4], with the resultant immunoassay demonstrating high sensitivity and specificity relative to the standard liquid chromatography/tandem mass spectrometry (LC-MS/MS) assay. Here, we further evaluate the immunoassay by comparing urine TFV measurements to TFV concentrations measured in plasma, which served as the gold standard metric for short-term adherence in most PrEP trials. Further, we assess the novel urine assay’s ability to predict protection from HIV in a large completed PrEP trial.

## METHODS

We used a randomly sampled, nested cohort of women and men, as well as cases of those who HIV seroconverted, assigned to the use of TFV disoproxil fumarate (TDF) or TDF/emtricitabine (FTC) in the Partners PrEP Study (NCT00557245), a randomized, placebo-controlled PrEP efficacy trial conducted among HIV serodiscordant couples in Kenya and Uganda [5]. During study follow-up, urine samples were archived at 3-, 12-, 24-, and 36-month visits. TFV concentrations were measured in all available archived urine samples using a quantitative enzyme-linked immunosorbent assay (ELISA; lower limit of quantification [LLOQ] = 1000 ng/mL) with this novel antibody [4]. TFV concentrations in date-matched plasma specimens were previously measured among the cohort using a validated LC-MS/MS assay (LLOQ = 0.31 ng/mL). TFV concentrations below the LLOQ were assigned a value of half the LLOQ for each respective assay. Urine and plasma samples were stored at −80^o^C upon collection and shipped on dry ice.

The correlation between paired urine and plasma concentrations was assessed, and the sensitivity and specificity of detectable TFV in urine for determining detectable TFV concentrations in plasma were calculated. Additionally, we determined the sensitivity and specificity of urine TFV levels ≥1500 ng/mL, a concentration indicative of PrEP dosing in the past day among Thai men and women [6], for determining plasma TFV concentrations >40 ng/mL, levels consistent with daily PrEP dosing [7].

To assess the association between urine TFV concentrations ≥1500 ng/mL and protection from HIV acquisition, we conducted a nested case-control analysis. Case samples collected on the date of first HIV-1 RNA detection were matched with control samples collected at the same study follow-up month. If the case’s first evidence of HIV was observed between regular urine sample archiving, controls from the nearest archive visit were selected. Control samples were matched 35:1, the ratio where estimates began to stabilize, and randomly sampled from the risk set of participants who were not living with HIV at the case’s date of HIV detection, including future seroconverters. Controls could be matched to multiple cases. Conditional logistic regression, adjusted for matched sets, estimated the odds ratio of HIV acquisition given a urine TFV concentration ≥1500 ng/mL, which approximates a rate ratio given our time-matched risk set sampling approach. Adjusted models controlled for participant sex, age, and report of any condomless sex with their study partner in the prior month at enrollment. All models were replicated to also assess the association of plasma TFV >40 ng/mL with HIV protection. Case samples were too few to conduct adequately powered sex-based subgroup analyses.

The protocol for the parent study received ethical approval from the University of Washington Institutional Review Board and ethics review committees at each study site. All participants provided written informed consent.

## RESULTS

Of 4432 individuals randomized to use of TDF or TDF/FTC in the Partners PrEP Study, 292 were included in the nested cohort. Among these participants, 39% were female and the median age was 33 years (interquartile range [IQR] = 28–39). Participants in the cohort contributed 722 paired urine and plasma samples. Of 52 individuals who seroconverted to HIV while using PrEP in the study, 22 had urine samples available from the visit where HIV was first detected and were included as cases. An additional 69 seroconverter samples collected prior to HIV infection were included as possible controls. Among cases, 55% were female and the median age was 33 years (IQR = 27–39).

The median duration from collection to assay of plasma and urine samples was 20 months and 103 months, respectively. In the cohort, the median TFV concentration was 37 500 ng/mL (IQR = 500–90 000) in urine via ELISA and 65.4 ng/mL (IQR = 1.6–103.0) in plasma via LC-MS/MS. The Spearman rank correlation coefficient (ρ) for the 2 measures was 0.46 (*P* < .001). Of 558 plasma samples with detectable TFV (≥LLOQ of 0.31 ng/mL), 486 had a paired urine sample with detectable TFV (≥LLOQ of 1000 ng/mL) for a sensitivity of 87% (95% confidence interval [CI] = 84%–90%). There were 164 plasma samples with undetectable TFV, of which 119 had a paired urine sample with undetectable TFV for a specificity of 73% (95% CI = 65%–79%). Of 468 individuals with plasma TFV >40 ng/mL, 420 had a paired urine sample with TFV ≥1500 ng/mL, for a sensitivity of 90% (95% CI = 87%–92%). Finally, 254 plasma samples had TFV levels ≤40 ng/mL, of which 146 had a paired urine sample with TFV <1500 ng/mL for a specificity of 57% (95% CI = 51%–64%).

In total, 770 control samples from 280 individuals were matched to the 22 case samples in our case-control study. Among participants in both active PrEP study arms, urine TFV ≥1500 ng/mL was associated with a 71% (95% CI = 30%–88%) reduction in HIV risk in the adjusted model (Table 1). By contrast, plasma TFV >40 ng/mL was associated with an 87% (95% CI = 54%–96%) reduction in HIV risk (Supplementary Table 1).

**Table 1.** Percent Human Immunodeficiency Virus Risk Reduction Associated With Urine Tenofovir Concentrations >1500 ng/mL as Measured by a Novel Immunoassay

n (%) With Urine Tenofovir ≥1500 ng/mL					
Case Samples: First Evidence of HIV	Control Samples	% HIV Risk Reduction^a^ (95% CI)	*P* Value	Adjusted % HIV Risk Reduction^a,b^ (95% CI)	Adjusted *P* Value^b^
8/22 (36)	527/770 (68)	73 (36–89)	.003	71 (30–88)	.006

Analyses include individuals assigned to tenofovir disoproxil fumarate (TDF)/emtricitabine or TDF-only pre-exposure prophylaxis. Estimates were generated using conditional logistic regression.

Abbreviations: CI, confidence interval; HIV, human immunodeficiency virus.

^a^Percent risk reduction calculated as follows: (1 – rate ratio) × 100.

^b^Adjusted for sex, age at enrollment, and report of any condomless sex with study partner in the month prior to enrollment.

## Discussion

The development of a low-cost POC assay to evaluate PrEP adherence would facilitate the implementation of real-time, drug-level feedback in current PrEP programs in resource-limited settings. Here, we demonstrate that urine TFV concentrations, measured using a novel immunoassay, predict protection from HIV acquisition among PrEP users in sub-Saharan Africa. The concentrations measured using the urine assay also had good sensitivity and specificity for plasma levels, the gold-standard metric of adherence in placebo-controlled PrEP trials.

Previous studies have highlighted the advantages of using a urine-based assay to evaluate PrEP use. TARGET, a pharmacokinetic study that randomized Thai adults to directly observed TDF in arms simulating low-, medium-, and high-adherence patterns, demonstrated that paired urine and plasma TFV concentrations measured by LC-MS/MS were highly correlated [8]. The study also showed that urine TFV concentrations can be used to evaluate time since dosing, further demonstrated in other US-based studies [9, 10]. Data from the United States have also suggested that urine collection is highly acceptable, which may result in high uptake compared with other biological measures [11]. Additional evidence regarding the feasibility and acceptability of urine-based measures of PrEP use in other settings, including sub-Saharan Africa, is needed.

Previously, we demonstrated that urine levels via this immunoassay were correlated with other biomarkers, including TFV and FTC levels in hair and TFV-diphosphate and FTC-triphosphate concentrations in DBS, among men who have sex with men and transwomen in the Pre-exposure Prophylaxis Initiative (iPrEX) open-label extension (OLE) study. Moreover, low urine concentrations among participants in iPrEx OLE were associated with subsequent HIV seroconversion [12]. The data presented here extend these prior results to heterosexual men and women on PrEP in sub-Saharan Africa.

There are several potential limitations to our results. First, we were unable to account for specific gravity of the urine samples or normalize to creatinine levels [9]. Second, longer storage of urine compared with plasma samples prior to analysis may have resulted in differential rates of sample degradation. Moderate correlation observed between urine and plasma TFV concentrations may be partially explained by these factors. Use of a specified TFV threshold for other analyses, however, may have mitigated the influence of measurement variability. Finally, most seroconverters did not have urine samples available from the visit of first HIV detection, limiting study power, and incomplete availability of control samples at urine archive months may have limited our control matching. However, our overall risk reduction estimates with plasma TFV >40 were nearly identical to those previously identified in this cohort [7], suggesting bias may have been minimal.

In summary, we show the high predictive utility of an adequate urine TFV level for HIV protection among men and women using PrEP in sub-Saharan Africa. The urine immunoassay has been developed into a lateral flow assay (LFA), which is low-cost, easy to perform, can be administered at the POC, and provides results within minutes [4]. The LFA is portable and requires no reagents, so it may also be administered in the field by nonmedical personnel to help reach stigmatized or hidden populations. The assay should be evaluated in a variety of populations for adherence monitoring and feedback.

## Supplementary Data

Supplementary materials are available at *Clinical Infectious Diseases* online. Consisting of data provided by the authors to benefit the reader, the posted materials are not copyedited and are the sole responsibility of the authors, so questions or comments should be addressed to the corresponding author.

ciaa785_suppl_Supplemental-Table-1

## Notes

**Partners PrEP study team:** University of Washington Coordinating Center and Central Laboratories, Seattle: Connie Celum (principal investigator, protocol cochair), Jared M. Baeten (medical director, protocol cochair), Deborah Donnell (protocol statistician), Robert W. Coombs, Jairam R. Lingappa, and M. Juliana McElrath.

**Study sites and site principal investigators:** Eldoret, Kenya (Moi University, Indiana University): Kenneth H. Fife, Edwin Were; Kabwohe, Uganda (Kabwohe Clinical Research Center): Elioda Tumwesigye; Jinja, Uganda (Makerere University, University of Washington): Patrick Ndase, Elly Katabira; Kampala, Uganda (Makerere University): Elly Katabira, Allan Ronald; Kisumu, Kenya (Kenya Medical Research Institute, University of California–San Francisco): Elizabeth Bukusi, Craig R. Cohen; Mbale, Uganda (AIDS Support Organization, Centers for Disease Control and Prevention (CDC)-Uganda): Jonathan Wangisi, James D. Campbell, Jordan W. Tappero; Nairobi, Kenya (University of Nairobi, University of Washington): James Kiarie, Carey Farquhar, Grace John-Stewart; Thika, Kenya (University of Nairobi, Kenya Medical Research Institute, University of Washington, Jomo Kenyatta University): Nelly R. Mugo, Kenneth Ngure; Tororo, Uganda (CDC-Uganda, AIDS Support Organization): James D. Campbell, Jordan W. Tappero, Jonathan Wangisi. Data management was provided by DF/Net Research, Inc (Seattle), and site laboratory oversight was provided by Contract Lab Services (University of the Witwatersrand, Johannesburg, South Africa).

***Acknowledgments.*** The authors thank all of the men and women who participated in the Partners PrEP (pre-exposure prophylaxis) Study as well as the clinical trial site teams.

***Financial support.*** This work and the development of the antibody at Abbott Rapid Diagnostics were supported by the National Institute of Allergy and Infectious Diseases (USAID)/National Institutes of Health (NIH; grant R01AI143340). W. C. R., G. W., and M. V. are from Abbott Rapid Diagnostics. R. M. S. was supported by an NIH institutional training grant (T32AI007140). M. A. S. was supported by an NIH institutional training grant (T32AI060530). Support for the Partners PrEP Study was provided by the Bill & Melinda Gates Foundation (OPP47674).

***Potential conflicts of interest.*** J. M. B. serves on advisory boards for Gilead Sciences, Merck, and Janssen and reports grants from USAID. D. V. G. serves on advisory boards for Gilead Sciences and Merck. W. C. R. is from Abbott Rapid Diagnostics, the developer of the antibody and has a patent pending. G. W. is from Abbott Rapid Diagnostics, the developer of the antibody. M. V. is from Abbott Rapid Diagnostics, the developer of the antibody. A. M. received nonfinancial support from Gilead Sciences.

M. M. received grants from the NIH outside the submitted work. C. H. received grants from Merck, Gilead Sciences, the NIH, and ViiV/GSK; personal fees from Merck and ViiV/GSK; nonfinancial support from Gilead Sciences; and has a patent issued on a tenofovir PrEP product. All other authors report no potential conflicts. All authors have submitted the ICMJE Form for Disclosure of Potential Conflicts of Interest. Conflicts that the editors consider relevant to the content of the manuscript have been disclosed.

## References

[1] Fonner VA, Dalglish SL, Kennedy CE, et al. Effectiveness and safety of oral HIV preexposure prophylaxis for all populations. AIDS 2016; 30:1973–83. 27149090 10.1097/QAD.0000000000001145 PMC4949005

[2] Yun K, Xu JJ, Zhang J, et al. Female and younger subjects have lower adherence in PrEP trials: a meta-analysis with implications for the uptake of PrEP service to prevent HIV. Sex Transm Infect 2018; 94:163–8. 28756409 10.1136/sextrans-2017-053217

[3] Haberer JE Current concepts for PrEP adherence in the PrEP revolution: from clinical trials to routine practice. Curr Opin HIV AIDS 2016; 11:10–7. 26633638 10.1097/COH.0000000000000220 PMC4801217

[4] Gandhi M, Wang G, King R, et al. Development and validation of the first point-of-care assay to objectively monitor adherence to HIV treatment and prevention in real-time in routine settings. AIDS 2019; 34:255–60. 10.1097/QAD.0000000000002395 PMC7021226 31634188

[5] Baeten JM, Donnell D, Ndase P, et al; Partners PrEP Study Team Antiretroviral prophylaxis for HIV prevention in heterosexual men and women. N Engl J Med 2012; 367:399–410. 22784037 10.1056/NEJMoa1108524 PMC3770474

[6] Gandhi M, Bacchetti P, Spinelli MA, et al. Brief report: validation of a urine tenofovir immunoassay for adherence monitoring to PrEP and ART and establishing the cutoff for a point-of-care test. J Acquir Immune Defic Syndr 2019; 81:72–7. 30664078 10.1097/QAI.0000000000001971 PMC6456396

[7] Donnell D, Baeten JM, Bumpus NN, et al. HIV protective efficacy and correlates of tenofovir blood concentrations in a clinical trial of PrEP for HIV prevention. J Acquir Immune Defic Syndr 2014; 66:340–8. 24784763 10.1097/QAI.0000000000000172 PMC4059553

[8] Drain PK, Kubiak RW, Siriprakaisil O, et al. Urine tenofovir concentrations correlate with plasma and relates to TDF adherence: a randomized directly-observed pharmacokinetic trial (TARGET Study). Clin Infect Dis 2019; 70:2143–51. 10.1093/cid/ciz645 PMC7201421 31314073

[9] Haaland RE, Martin A, Livermont T, et al. Urine emtricitabine and tenofovir concentrations provide markers of recent antiretroviral drug exposure among HIV-negative men who have sex with men. J Acquir Immune Defic Syndr 2019; 82:252–6. 31335590 10.1097/QAI.0000000000002133 PMC7542201

[10] Koenig HC, Mounzer K, Daughtridge GW, et al. Urine assay for tenofovir to monitor adherence in real time to tenofovir disoproxil fumarate/emtricitabine as pre-exposure prophylaxis. HIV Med 2017; 18:412–8. 28444867 10.1111/hiv.12518 PMC5459623

[11] Hunt T, Lalley-Chareczko L, Daughtridge G, Swyryn M, Koenig H Challenges to PrEP use and perceptions of urine tenofovir adherence monitoring reported by individuals on PrEP. AIDS Care 2019; 31:1203–6. 30821473 10.1080/09540121.2019.1587369

[12] Spinelli MA, Glidden DV, Rodrigues WC, et al. Low tenofovir level in urine by a novel immunoassay is associated with seroconversion in a preexposure prophylaxis demonstration project. AIDS 2019; 33:867–72. 30649051 10.1097/QAD.0000000000002135 PMC6375797

