# Supplementary material for: Urine Tenofovir Levels Measured Using a Novel Immunoassay Predict Human Immunodeficiency Virus Protection
Source: Clin Infect Dis. 2020 Jun 22;72(3):486–9. doi: 10.1093/cid/ciaa785 (PMC7850546; doi:10.1093/cid/ciaa785)
Supplement: ciaa785_suppl_Supplemental-Table-1 [file ciaa785_suppl_supplemental-table-1.docx]

**Supplemental Table 1**. Percent HIV risk reduction associated with plasma concentrations >40 ng/mL as measured by a liquid chromatography-tandem mass spectrometry method

| n (%) with plasma TFV >40 ng/mL | |  |  |  |  |
| --- | --- | --- | --- | --- | --- |
| Case samples:  First evidence of HIV | **Control samples** | **% HIV risk reduction**^a^  **(95% CI)** | **p-value** | **Adjusted % HIV risk reduction**^a,b^  **(95% CI)** | **Adjusted p-value**^b^ |
| 3/22 (14%) | 461/770 (60%) | 89% (64 to 97%) | <0.001 | 87% (54 to 96%) | 0.002 |
| TFV: tenofovir, CI: confidence interval  Analyses include individuals assigned to TDF/FTC or TDF-only PrEP. Estimates were generated using conditional logistic regression.  ^a^% risk reduction calculated as follows: (1-RR)*100  ^b^Adjusted for sex, age at enrollment, and report of any condomless sex with study partner in the month prior to enrollment | | | | | |
